# Ultrasound-Guided Subclavian Vein Catheterization: Contrasting Supraclavicular and Infraclavicular Approaches for Enhanced Procedural Precision

**DOI:** 10.7759/cureus.60974

**Published:** 2024-05-24

**Authors:** Rama Devi

**Affiliations:** 1 Anaesthesiology, Velammal Medical College and Research Institute, Madurai, IND

**Keywords:** central venous access, infraclavicular, supraclavicular, catheterization, subclavian vein, ultrasound-guided

## Abstract

Background

Ultrasound-guided subclavian vein catheterization is crucial for central venous access, but the choice between the supraclavicular and infraclavicular approaches lacks comprehensive comparison. This study addresses this gap by conducting a prospective observational analysis of both techniques. The supraclavicular method accesses the vein from above the clavicle while the infraclavicular targets it below. Our model-driven approach aims to elucidate the procedural nuances, success rates, and complications associated with each method. The findings intend to equip clinicians with evidence-based insights, facilitating informed decision-making for improved procedural outcomes in ultrasound-guided subclavian vein catheterization.

Aim and objective

This study aims to comprehensively compare the supraclavicular and infraclavicular approaches in ultrasound-guided subclavian vein catheterization, evaluating the procedural minutiae, potential advantages, and challenges associated with each technique. Employing a prospective observational methodology, our objective is to provide evidence-based insights for approaches in ultrasound-guided subclavian vein catheterization, evaluating procedural nuances, success rates, and complications during the procedure.

Methods

In this prospective investigation, 276 patients aged between 20 and 55 years were randomly assigned to two groups: 143 patients in the supraclavicular group and 133 patients in the infraclavicular group. Specifically, patients admitted for elective surgery necessitating postoperative ICU care were considered. The study assessed various variables, including success rate, time required for venous visualization, venous puncture, catheterization, total procedure duration, and incidence of mechanical complications, to facilitate group comparisons.

Results

The mean procedural time was shorter in the supraclavicular group compared to the infraclavicular group, with durations of 2 minutes and 2 seconds versus 3 minutes and 40 seconds, respectively (95% CI). This difference was statistically significant. Similarly, the mean durations for venous visualization, venous puncture, and venous catheterization were also shorter in the supraclavicular group, and these differences were statistically significant. Both groups achieved a 100% success rate, with the first attempt success rate being higher in the supraclavicular subclavian vein group.

Conclusion

The findings of this study demonstrate a statistically significant advantage in favor of the supraclavicular approach for ultrasound-guided subclavian vein catheterization. The shorter mean procedural time, as well as durations for venous visualization, puncture, and catheterization, emphasize the efficiency of the supraclavicular technique. The consistently achieved 100% success rate, coupled with a higher first-attempt success rate, further underscores the proficiency of the supraclavicular subclavian vein group. These results collectively suggest that the supraclavicular approach is not only time-efficient but also superior in terms of successful central line placement, making it a promising choice for both emergency and critical care settings.

## Introduction

The digital revolution in India has catalyzed a paradigm shift in healthcare delivery, ushering in an era marked by the widespread adoption of modernized medical equipment. Amidst this transformative landscape, the demand for advanced interventions in critical care settings has surged, with central venous catheterization emerging as a cornerstone procedure in optimizing patient management. Statistics from 2022 from the website of global data, the use of the central vein has increased by upto 2 million in India, a significant reliance on central venous access for delivering optimal care to critically ill patients, highlighting the procedure's pivotal role in contemporary healthcare [1,2].

The infraclavicular approach to accessing the subclavian vein has been traditionally favored in central venous catheterization. However, recent research has illuminated the potential advantages offered by the supraclavicular approach. Studies have showcased notable enhancements in success rates and safety profiles associated with the supraclavicular method [2,3]. Leveraging the power of ultrasound guidance has emerged as a promising avenue to refine subclavian access techniques, intending to mitigate the complications often encountered with the infraclavicular approach [4].

This study highlights the comparative efficacy of the infraclavicular and supraclavicular approaches in accessing the subclavian vein during central venous catheterization. By carefully examining the details surrounding ultrasound-guided subclavian access and a juxtaposition with traditional landmark techniques, our goal is to ascertain whether the supraclavicular approach is genuinely superior to the infraclavicular method, or if the traditional infraclavicular approach still holds sway in subclavian access. By undertaking this investigation, we aim to elucidate strategies for optimizing patient outcomes in critical care settings.

Numerous articles and studies will inform our exploration, including research on the success rates and safety profiles associated with each approach and comparative analyses of complication rates and patient outcomes [1,3,5]. These sources will serve as the foundation for our study, providing robust evidence to support our findings and recommendations. Through rigorous investigation and analysis, we aim to contribute valuable insights to the field of central venous catheterization, ultimately enhancing the quality of care delivered to critically ill patients.

## Materials and methods

This prospective observational study aims to assess the efficacy and safety of ultrasound-guided subclavian vein catheterization, comparing the supraclavicular and infraclavicular approaches. After getting approval from the Institutional Research and Ethical Committee (Velammal Medical College and Research Institute)clearance Ref: IEC No. VMCHIEC/06/2021, the study was conducted at the main Operation Theater (OT) complex in Velammal Medical College Hospital and Research Institute from March 2022 to December 2023. Sample size calculations were based on the anticipated difference in success rates between the two approaches, aiming for a power of 80%. Adult patients aged 18 years and above requiring subclavian vein catheterization for medical or surgical indications, with a preoperative body mass index (BMI) ranging from 18.5 to 40 kg/m² calculated using the metric formula: weight (kg)/[height (m)]^2^, and who provided signed informed consent are included. The overall sample population was 380. We excluded 110 patients based on our exclusion criteria, which encompassed patients with known subclavian vein thrombosis or obstruction, severe coagulopathy or bleeding disorders, significant anatomical abnormalities in the subclavian region, inability to provide informed consent or cooperate with the procedure, and contraindications to ultrasound-guided procedures (e.g., allergy to ultrasound gel). Convenience sampling to assign eligible patients to either the supraclavicular or infraclavicular group [6,7]. Materials used are 7Fr multiple lumen central venous catheter 16,18 G with 16 cm catheter length, an ultrasound machine with a linear probe with frequency 6 to 13 MH.

Experienced anesthetists/operators performed ultrasound-guided subclavian vein catheterization using either the supraclavicular or infraclavicular approach. The choice of catheter size and brand was left to the discretion of the anesthetists/operators.

Outcome measures

The primary outcome was the success rate of subclavian vein catheterization on the first attempt. Secondary outcomes included the number of needle passes required for successful catheterization, time taken for the procedure, incidence of complications (e.g., pneumothorax, hematoma, arterial puncture), patient discomfort during the procedure (assessed using a visual analog scale), and operator satisfaction (measured using a Likert scale) [8].

## Results

Data analysis was conducted using SPSS software. Appropriate statistical tests such as the chi-square test, t-test, or Mann-Whitney U test were applied, depending on the data type, with a significance level set at p < 0.05.

Data were entered into Microsoft Excel 2017 (Microsoft Corporation, Redmond, WA, US) and data analysis was performed using Statistical Package for the Social Sciences for Windows (SPSS) version 20 (IBM Corp., Armonk, NY, US). Frequency and percentages were utilized to express the categorical variables while mean and standard deviation were employed for continuous variables. To compare the two groups, the student t-test was utilized to measure statistical significance between the two groups, with a p-value of < 0.05 considered statistically significant.

The primary outcome was the success rate of subclavian vein catheterization on the first attempt. Secondary outcomes included the number of needle passes required for successful catheterization, time taken for the procedure, incidence of complications (e.g., pneumothorax, hematoma, arterial puncture), patient discomfort during the procedure (assessed using a visual analog scale), and operator satisfaction (measured using a Likert scale((1) Strongly Disagree; (2) Disagree; (3) Neither Agree nor Disagree; (4) Agree; (5) Strongly Agree).

The demographic parameters such as age were comparable between the two groups and statistical association was done and showed no significance and the p-value was 0.731. The distribution of study participants based on their BMI, patients with normal weight were found to be 114 (79.7%), overweight were 20 (13.9%), and only 4.3% of the study participants fell underweight in the supraclavicular group and were found to be statistically insignificant when compared with infraclavicular group (Table 1).

**Table 1:** Distribution of basic characteristics between two groups

Basic characteristic	Supraclavicular approach (mean ± SD)	Infraclavicular approach (mean ± SD)	P value
Age (in years)	43.5 ± 11.7	46 ± 7.5	0.731
Males	93 (65%)	91 (68%)	0.233
Underweight	9 (4.3%)	6 (4.5%)	0.101
Normal weight	114 (79.7%)	120 (90.2%)	0.345
Overweight	20 (13.9%)	10 (7.5%)	0.023

Total procedural time (mean ± SD) was less in the supraclavicular group than in the infraclavicular group (120.6 ± 20 vs 180 ± 40 seconds with 95% CI) and the difference was statically significant with a p-value of 0.001. Similarly, venus visualization, venous puncture, and venous catheterization of mean ± SD were less in the supraclavicular than in the infraclavicular group and showed statistical significance (Table 2). Table 3 and Table 4 list the differences in outcome measures, patient’s pain visual analog scale, and operator’s Likert scale between the supraclavicular and infraclavicular groups.

**Table 2:** Difference in procedural time between the supraclavicular and infraclavicular groups *p-value <0.05 shows statistical significance

Time (in seconds)	Supraclavicular approach time (in seconds) (mean ± SD)	Infraclavicular approach time (in seconds) (mean ± SD)	P-value
Venous visualization	13 ± 2	25 ± 3	0.0001*
Venous puncture	34 ± 4	50 ± 4	0.003*
Venous catheterization	121 ± 4	138 ± 5	0.015*

**Table 3:** Comparison between the supraclavicular and infraclavicular groups in terms of outcome measures *A p-value<0.05 was considered to be statistically significant.

Outcome measures	Supraclavicular (n=143)	Infraclavicular (n=133)	P-value
Second attempt	5 (3.5%)	11 (8.3%)	0.081
Reposition	3 (2%)	13 (9.7%)	0.091
Arterial injury	4 (2.8%)	5 (3.8%)	0.231
Hematoma	5 (3.5%)	17 (12.8%)	0.05
Pneumothorax	0 (0)	1 (0.8%)	0.456
Arrhythmia	7 (4.9%)	27 (20%)	0.0421*
Catheter blockage	9 (6.3%)	53 (40%)	0.05
Probe to vein distance	1.9 ± 1.1 cm	2.5 ± 1.5 cm	0.032*

**Table 4:** Comparison of patient’s pain visual analog scale and operator’s Likert scale between the supraclavicular and infraclavicular groups *p-value <0.05 was considered to be statistically significant. Outcome measures in terms of patient’s pain visual analog scale (0-10)and operator’s Likert scale (1-5) scores in numbers.

Outcome measures (in SCORES)	Supraclavicular (n=143)	Infraclavicular (n=133)	P value
Patient’s pain visual analog scale	4-5 (89%)	6-9 (75%)	0.04*
Operator’s Likert scale	5 (85 %)	4 (77%)	0.03*

## Discussion

The study findings align with existing literature, supporting the advantages of the supraclavicular approach for subclavian vein catheterization. Anatomically, this approach provides more direct and superficial access to the subclavian vein compared to the infraclavicular approach, minimizing the risk of complications associated with inadvertent puncture of surrounding structures [9]. Studies consistently demonstrate shorter procedural times and higher success rates with the supraclavicular approach, attributed to improved visualization and precise needle placement facilitated by real-time ultrasound guidance [10].

Sanini et al. supported our result in terms of outcomes, the supraclavicular approach exhibits superior performance, with lower rates of complications such as arterial puncture, hematoma, and pneumothorax compared to the infraclavicular approach [6]. Additionally, higher catheter patency rates and lower rates of catheter-related infections are associated with the supraclavicular approach.

In our study, patient and operator satisfaction was also higher with the supraclavicular approach, attributed to a streamlined procedural experience and improved catheter performance. This positive feedback underscores the clinical benefits of the supraclavicular approach in improving patient safety and reducing healthcare-associated costs.

Jain et al.'s retrospective study over one year demonstrated the ease, safety, and practical convenience of the supraclavicular approach, with a significant correlation between acute complications and insertion position favoring the supraclavicular approach [11]. Similarly, Czarnik's study prospective cohort study emphasized the excellent success rate and low complication rate of subclavian venous catheterization via the supraclavicular approach, particularly in mechanically ventilated patients [12].

Byon's prospective, randomized study in children under three years old showed shorter puncture times and decreased incidences of guidewire misplacement with the supraclavicular approach [13]. Fragou's randomized controlled trial demonstrated a significantly lower rate of catheterization-related complications with the supraclavicular approach compared to the infraclavicular approach [9].

Parienti's meta-analysis supported the superiority of the supraclavicular approach in terms of lower failure rates and decreased incidence of catheter malposition [14]. Stachura's prospective survey highlighted a better ultrasound view of the subclavian vein with the supraclavicular approach [15].

**Figure 1:**
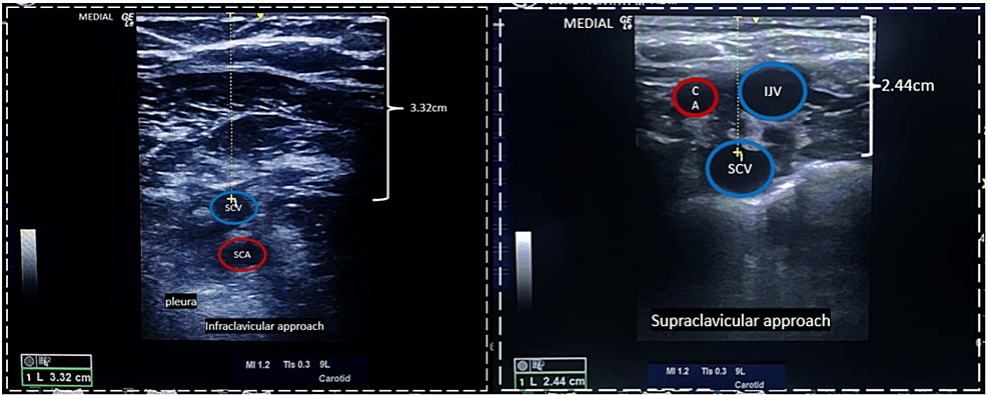
Anatomical marking from the probe to the vein in real-time ultrasound showing the distance difference between the infraclavicular and supraclavicular approach of subclavian vein access The anatomical landmark of the subclavian vein; the distance between the probe and the subclavian vein in the infraclavicular approach is 3.32 cm. The distance between the probe and the subclavian vein in the supraclavicular approach is 2.44 cm. Red color marking (SCA: subclavian artery and CA: carotid artery); blue color marking (IJV: internal jugular vein and SCV: subclavian vein) (original image)

In a real-time ultrasound study comparing the anatomical markings from probe to vein, it was found that the supraclavicular approach to subclavian vein access demonstrated a shorter distance of 2.44 cm compared to the infraclavicular approach's 3.32 cm (Figure 1). This suggests that the subclavian vein is more superficially located in the supraclavicular region, making it easier to access and handle complications such as hematoma formation. The increased subcutaneous fatty tissue in obese patients further complicates infraclavicular access, making the supraclavicular approach more promising for ease of subclavian vein access. This finding underscores the potential advantages of utilizing the supraclavicular approach in clinical practice.

While the evidence overwhelmingly supports the benefits of the supraclavicular approach, it's important to acknowledge potential limitations and complications associated with central venous catheterization. Studies have reported technical complications, such as failure attempts, pneumothorax, arterial puncture, and catheter embolization, underscoring the need for proper training and adherence to guidelines [5].

The main limitations of this study are the small sample size, single-centered study, and operator blinding. These limitations underscore the need for larger, multicenter studies with diverse patient populations, standardized protocols, and rigorous blinding procedures to ensure robust and unbiased conclusions regarding the comparative efficacy of different venous catheterization approaches. There is no conflict of interest.

## Conclusions

In conclusion, our study confirms the superior efficacy of the supraclavicular approach for subclavian vein catheterization compared to the infraclavicular approach. Through shorter procedural times, enhanced visualization, and higher success rates, this approach minimizes complications and improves patient outcomes. These findings align with existing literature, emphasizing the anatomical and procedural advantages of the supraclavicular approach. Despite its benefits, careful training and adherence to guidelines remain essential to mitigate potential risks. Overall, our study underscores the importance of adopting the supraclavicular approach as the preferred method for central venous catheterization, offering clinicians a reliable and efficient technique that enhances patient safety and satisfaction. Continued research and education are vital to further refine and standardize the use of this approach, ensuring its widespread adoption and positive impact on clinical practice.
